# Compound heterozygous alterations in intraflagellar transport protein *CLUAP1* in a child with a novel Joubert and oral–facial–digital overlap syndrome

**DOI:** 10.1101/mcs.a001321

**Published:** 2017-07

**Authors:** Jennifer J. Johnston, Chanjae Lee, Ingrid M. Wentzensen, Melissa A. Parisi, Molly M. Crenshaw, Julie C. Sapp, Jeffrey M. Gross, John B. Wallingford, Leslie G. Biesecker

**Affiliations:** 1 Medical Genomics and Metabolic Genetics Branch, National Human Genome Research Institute, National Institutes of Health, Bethesda, Maryland 20892-4472, USA;; 2 Department of Molecular Biosciences, University of Texas at Austin, Austin, Texas 78705, USA;; 3 Intellectual and Developmental Disabilities Branch, *Eunice Kennedy Shriver* National Institute of Child Health and Human Development, National Institutes of Health, Bethesda, Maryland 20892, USA;; 4 Departments of Ophthalmology and Developmental Biology, University of Pittsburgh, Pittsburgh, Pennsylvania 15260, USA

**Keywords:** 2–3 toe syndactyly, abnormality of the eyebrow, accessory oral frenulum, broad nasal tip, central hypotonia, cutaneous finger syndactyly, depressed nasal bridge, dysgenesis of the cerebellar vermis, high, narrow palate, hypertelorism, incomplete cleft of the upper lip, mixed hearing impairment, patent foramen ovale, postaxial hand polydactyly, preaxial foot polydactyly, prominent epicanthal folds, rhizomelic arm shortening, sparse scalp hair, thick anterior alveolar ridges, underdeveloped supraorbital ridges

## Abstract

Disruption of normal ciliary function results in a range of diseases collectively referred to as ciliopathies. Here we report a child with a phenotype that overlapped with Joubert, oral–facial–digital, and Pallister–Hall syndromes including brain, limb, and craniofacial anomalies. We performed exome-sequence analysis on a proband and both parents, filtered for putative causative variants, and Sanger-verified variants of interest. Identified variants in *CLUAP1* were functionally analyzed in a *Xenopus* system to determine their effect on ciliary function. Two variants in *CLUAP1* were identified through exome-sequence analysis, Chr16:g.3558407T>G, c.338T>G, p.(Met113Arg) and Chr16:g.3570011C>T, c.688C>T, p.(Arg230Ter). These variants were rare in the Exome Aggregation Consortium (ExAC) data set of 65,000 individuals (one and two occurrences, respectively). Transfection of mutant *CLUAP1* constructs into *Xenopus* embryos showed reduced protein levels p.(Arg230Ter) and reduced intraflagellar transport p.(Met113Arg). The genetic data show that these variants are present in an affected child, are rare in the population, and result in reduced, but not absent, intraflagellar transport. We conclude that biallelic mutations in *CLUAP1* resulted in this novel ciliopathy syndrome in the proband.

## INTRODUCTION

Human ciliopathies arise from mutation in any of dozens of genes implicated in ciliogenesis, and many of these genes are grouped in functional units. One such unit is formed by the intraflagellar transport (IFT) proteins, which collaborate to move cargo into and out of cilia (Lechtreck 2015). Mutations in several IFT genes have been implicated previously in diverse ciliopathies (Beales et al. 2007; Arts et al. 2011; Boldt et al. 2011), as have proteins controlling recruitment and assembly of IFT particles (Toriyama et al. 2016). CLUAP1 is a relatively recently described member of the IFT complex, having been shown to be involved in ciliogenesis in mice, zebrafish, and the frog *Xenopus* (Pasek et al. 2012; Botilde et al. 2013; Lee et al. 2014). Recently, hypomorphic mutations were identified in human *CLUAP1* associated with the retinal ciliopathy Leber congenital amaurosis (Soens et al. 2016).

Here, we expand the role of CLUAP1 in the ciliopathy spectrum by describing a proband originally enrolled in a study of the genetic etiology of polydactyly with additional associated features. Exome sequencing identified compound heterozygous mutations in *CLUAP1*, and functional analysis on the altered protein in the *Xenopus* model of intraflagellar transport demonstrates that these alleles disrupt the normal function of CLUAP1.

## RESULTS

### Clinical Presentation and Family History

**Figure 1.**
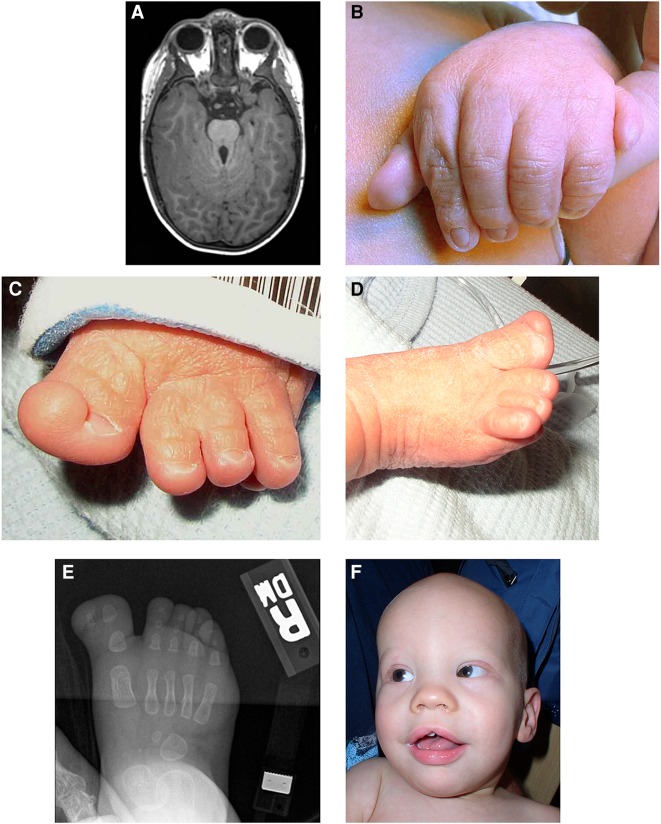
Patient images showing (*A*) (via magnetic resonance imaging) the molar tooth sign; (*B*) the left hand showing postaxial polydactyly and partial cutaneous syndactyly of the fourth and fifth digits; (*C*) the left foot showing partial duplication of the hallux and partial cutaneous syndactyly of the second and third toes; (*D*) the right foot showing the broad hallux and nail; (*E*) (via radiograph) the right foot showing the duplicated distal phalanx of the hallux; and (*F*) the proband as an infant showing sparse scalp hair and eyebrows, epicanthal folds, wide nasal bridge and tip, and midline notch in the upper lip.

The proband was born at 36 wk gestation, the second pregnancy of a 27-yr-old female. On examination at 3 d, he had sparse scalp hair and eyebrows, underdeveloped supraorbital ridges, apparently widely spaced eyes with epicanthal folds, a wide and mildly depressed nasal bridge, a broad nasal tip, and retrognathia. His oral findings included a midline notched upper lip, alveolar ridge overgrowth, high palate, extra frenula, a malformed epiglottis with a midline cleft, and a notched tongue tip (Fig. 1F). His limb findings were remarkable for mild rhizomelic shortening, bilateral postaxial polydactyly with partial cutaneous syndactyly of fingers 4–5, bilateral preaxial polydactyly with partial cutaneous syndactyly of toes 2–3, broadened metatarsals, short fingers and toes, and small nails (Fig. 1B–E). Additionally, a patent foramen ovale, breathing difficulties, and hypotonia were noted. He had mild bilateral mixed hearing loss, for which he had tympanostomy tubes and hearing aids placed at the age of 1 yr. At the age of 2 yr, magnetic resonance imaging (MRI) showed a mildly to moderately small cerebellar vermis, horizontal and thick superior cerebellar peduncles, superior cerebellar dysplasia, and cerebellar tonsil dysplasia, consistent with the molar tooth sign (Fig. 1A). Also at the age of 2 yr, a gastrostomy tube (G tube) was placed for swallowing difficulty due to moderate pharyngeal phase dysphagia. At the age of 5 yr, a small penis and disproportionate rhizomelic shortening in the upper and lower limbs were noted. An electroencephalography for recurrent seizure episodes showed mild excessive background slowing for age; the episodes were described as petit mal or absence seizures possibly of frontotemporal origin. At this time, hypodontia, midline supernumerary tooth, fused teeth, narrow frenulum, a prominent upper lip, recurrent otitis media with effusion, chronic hearing loss likely mixed in nature, crowded oropharynx space (Mallampati type III), and tonsils 2–3+ in size were noted. At 9 yr, mild obstructive sleep apnea was diagnosed because of short stature, an increased body mass index (BMI) (see below for growth parameters), a small airway, redundant arytenoids, and hypotonia. At the age of 10 yr, he was noted to have a prominent epiglottis, a small larynx, and a bone age of 8–9 yr (mildly delayed). Growth parameters included an occipital–frontal circumference (OFC) of 55.2 cm (90th centile), height of 127.6 cm (2nd centile), weight of 45.6 g (92nd centile), and BMI of 28.0 (99th centile, obese). He had global developmental delay with oculomotor apraxia and drew pictures appropriate for a 4 yr old. He had a speech delay with poor volume and articulation. His family history was unremarkable, and there was no consanguinity reported. Genetic testing was negative for *MKS1, RPGRIP1L, AHI1, CEP290, TMEM67/MKS3, GLI3, NPHP1, CC2D2A, TMEM216,* and *OFD1*. He was felt to have a phenotype that overlapped Joubert syndrome, oral–facial–digital syndromes (OFDSs), and Pallister–Hall syndrome (Table 1) (Hall et al. 1980; Parisi and Glass 1993; Romani et al. 2013; Valente et al. 2013; Franco and Thauvin-Robinet 2016).

**Table 1.** Clinical findings in the present patient compared with Joubert syndrome, oral–facial–digital syndrome (OFDS) type II, and Pallister–Hall syndrome

	Proband	Joubert syndrome^a^	OFDS type II	Pallister–Hall syndrome
Widely spaced eyes or dystopia canthorum	+	(+)	+	−
Tongue clefts or hamartomas	Hamartomas	(+) Clefts or hamartomas	+Clefts	(+) Hamartomas
Abnormal oral frenula	Numerous	(+)	Numerous or thick	(+) Numerous
Abnormal dentition	+	−	(+)	−
Midline cleft lip	+	(+)	+	−
Cleft palate	−	(+)	(+)	−
Epiglottis bifid/cleft	+	−	−	+
Polydactyly	Postaxial hands and preaxial of toes	(+) Variable pre and postaxial	Postaxial hands and preaxial of toes	Mesoaxial or postaxial
Cutaneous syndactyly	Involving duplicated halluces	(+)	Involving duplicated halluces	+
Short fingers	−	−	(+)	+
Short limbs	+	−	(+)	−
Cystic/dysplastic kidneys	−	(+)	−	(+)
Liver fibrosis	−	(+)	−	−
Retinal dystrophy	−	(+)	−	−
Leber congenital amaurosis	−	(+)	−	−
Intellectual disability	+	+	(+)	−
Hypotonia	+	+	(+)	−
Oculomotor apraxia	+	+	(+)	−
Breathing abnormalities	+	+	(+)	−
Molar tooth sign	+	+	−	−
Encephalocele	−	(+)	(+)	−
Polymicrogyria	−	(+)	−	−
Hypothalamic hamartoma	−	−	(+)	+
Porencephaly	−	−	(+)	−
Hydrocephalus	−	(+)	(+)	−

+, Typically present; (+) present but not consistent; −, not typically present.

^a^Joubert syndrome is genetically and clinically heterogeneous.

### Exome Analysis

No variants passed our filtering criteria for either a de novo or autosomal recessive homozygous inheritance model. Variants were identified in two genes, *LRRC4* and *CLUAP1,* which fit an autosomal recessive compound heterozygous model. LRRC4 is suggested to have a role in tumor progression (Zhang et al. 2005). Based on the role of CLUAP1 in ciliogenesis during embryonic development in model animals (Pasek et al. 2012; Botilde et al. 2013; Lee et al. 2014), we concluded that the variants in *CLUAP1* were more likely to account for the proband's phenotype. Variants, Chr16:g.3558407T>G c.338T>G, p.(Met113Arg) and Chr16:g.3570011C>T, c.688C>T, p.(Arg230Ter) (reference cDNA NM_015041.2) (Table 2), were Sanger-verified in the trio, with both variants present in the proband, c.338T>C in the father and c.688C>T in the mother. Both variants were rare in the Exome Aggregation Consortium (ExAC; http://exac.broadinstitute.org) data set of 65,000 individuals (one and two occurrences, respectively) (Lek et al. 2016).

**Table 2.** Variant table

Gene	Chrom	DNA variant hg19/GRC37	HGVS protein Ref.	Variant type	Predicted effect	dbSNP/dbVar ID	Genotype proband	Genotype mother	Genotype father	ClinVar ID
*CLUAP1*	16	NC_000016.9: g.3570011C>T	XP_005255243.1:p.Arg230Ter	SNV; Nonsense	p.(Arg230Ter)	rs769705065	CT	CT	CC	SCV000299272
						Coverage:	26	28	35	
*CLUAP1*	16	NC_000016.9: g.3558407T>G	XP_005255243.1:p.Met113Arg	SNV; Missense	p.(Met113Arg)	rs768663992	GT	TT	GT	SCV000299273
						Coverage:	28	40	31	

HGVS, Human Genome Variation Society; dbSNP, Database for Short Genetic Variations; dbVar, Database of Genomic Structural Variation; SNV, single-nucleotide variant.

### Intraflagellar Transport and Localization Studies

**Figure 2.**
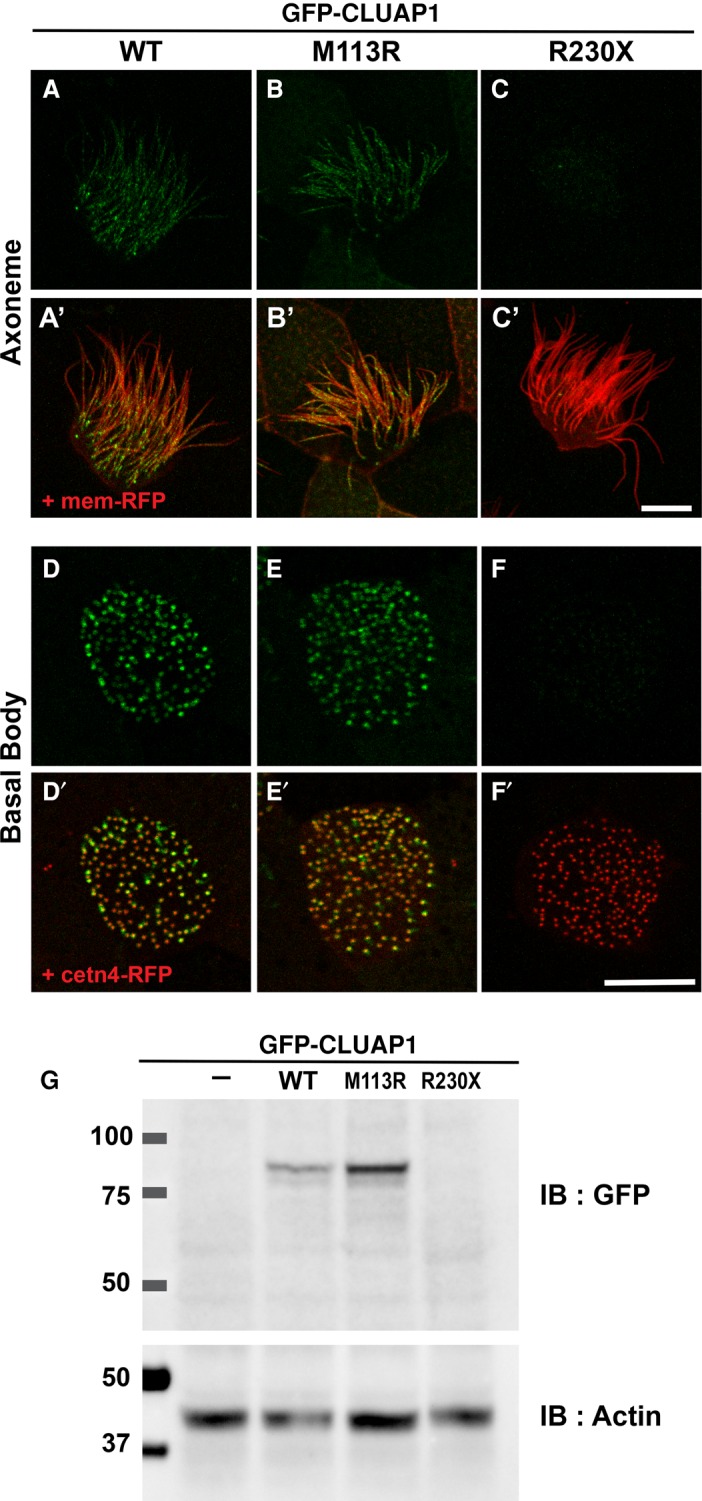
The *CLUAP1* M113R mutant is expressed and localized normally, whereas the expression level of R230X mutants is extremely low. (*A*–*F*) Live images displaying localization of green fluorescent protein (GFP)-tagged CLUAP1 WT, M113R, and R230X proteins at the axonemes (*A*–*C*) and basal bodies (*D*–*H*) in *Xenopus* multiciliated cells. (*A*′–*C*′) Merge views with membrane red fluorescent protein (RFP) visualizing axonemes. (*D*′–*F*′) Merge views with cetn4-RFP, a basal body marker. Scale bars, 10 µm. (*G*) Western blotting of GFP-tagged CLUAP1 proteins. Actin was used as a loading control.

We used expression of green fluorescent protein (GFP)-tagged mutant CLUAP1 proteins in *Xenopus* embryos to assess the effect of the variants on localization and intraflagellar transport. The p.(Met113Arg) variant was localized normally to both the axoneme and the basal body (Fig. 2A,B,D,E). In contrast, the nonsense mutation, p.(Arg230Ter), resulted in extremely low protein levels (Fig. 2C,F), a result that was confirmed by western blotting (Fig. 2G), so no further assessment was possible. Western blotting revealed that p.(Met113Arg) was expressed at levels comparable to wild-type Cluap1 in *Xenopus* embryos.

**Figure 3.**
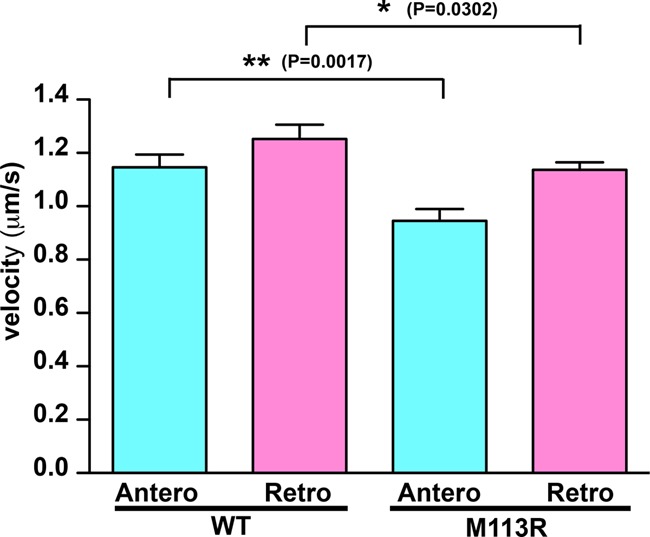
The *CLUAP1* M113R mutant has significantly reduced intraflagellar transport (IFT) velocities. The graph shows velocities of green fluorescent protein (GFP)-tagged CLUAP1 wild type (WT) and M113R. Mean velocities ± SEM of CLUAP1 WT are 1.15 ± 0.05 µm/sec for anterograde (*N* = 33) and 1.25 ± 0.05 µm/sec for retrograde (*N* = 61). Mean velocities ± SEM of CLUAP1 M113R are 0.95 ± 0.05 µm/sec for anterograde (*N* = 42) and 1.14 ± 0.03 µm/sec for retrograde (*N* = 76). Comparing with WT, IFT velocities of M113R are significantly reduced (*P* value of WT vs. M113R is 0.0017 for anterograde and 0.0302 for retrograde).

Because the p.(Met113Arg) variant was expressed and localized normally, we turned to high-speed confocal imaging of IFT movements to determine whether this variant had a more subtle defect. Indeed, we found that the average velocity for both anterograde and retrograde transport were reduced for the mutant when compared with wild type (Fig. 3). Though relatively minor, these reductions in IFT rate were comparable to those associated with defective ciliogenesis in previous studies (Tran et al. 2008; Chung et al. 2014). Together, our data suggest that the p.(Arg230Ter) truncation is likely a null allele, whereas the p.(Met113Arg) is likely hypomorphic.

## DISCUSSION

Disorders of ciliary function are collectively known as ciliopathies and include diseases that affect single organ systems to multisystemic developmental disorders including polycystic kidney disease, retinal dystrophy, Meckel syndrome, Joubert syndrome, OFDS, and others (Ferkol and Leigh 2012). IFT, the active movement of proteins within the cilium, plays a primary role in the formation of the cilia and cilia-mediated signaling. Disruption of this process through mutations in a number of genes that code for ciliary components causes ciliopathies (Lechtreck 2015). CLUAP1 has been identified as a component of the IFT complex B and is believed to regulate the IFT cycle at the base and tip of the cilium. Recently, a single proband has been described with Leber congenital amaurosis with a homozygous missense mutation in *CLUAP1*, c.817C>T; p.(Leu273Phe), supporting the role of CLUAP1 in ciliary function (Soens et al. 2016). Here we report the identification of compound heterozygous mutations in *CLUAP1* in a proband with a phenotype that included limb, central nervous system (CNS), and craniofacial anomalies that overlapped with Joubert syndrome, OFDS, and Pallister–Hall syndrome, thus expanding the understanding of *CLUAP1* in disease.

The implication of CLUAP1 in ciliopathies is supported by diverse data from model animals. For example, zebrafish *au5* mutants demonstrate photoreceptor degeneration, embryonic lethality, and defects of ciliogenesis (Lee et al. 2014) owing to a nonsense mutation in *cluap1.* Likewise, knockout of *Cluap1* in mice leads to embryonic lethality with abnormal heart looping and embryonic turning (Botilde et al. 2013), consistent with early defects in ciliogenesis. The proband presented here was compound heterozygous for a nonsense, p.(Arg230Ter), and a missense, p.(Met113Arg), mutation in *CLUAP1*. The nonsense mutation should result in limited protein production, presumably because of nonsense-mediated decay, whereas the missense mutation is expected to be hypomorphic, allowing for sufficient CLUAP1 function to escape embryonic lethality. Indeed, the p.(Met113Arg) mutation produced similar levels of protein when compared with wild type, but this p.(Met113Arg) mutant demonstrated slower IFT transport when compared with wild-type protein. *TTC21B* is another ciliary gene, mutations in which have been shown to cause nephronophthisis type 12 (OMIM [Online Mendelian Inheritance in Man; http://www.omim.org/] 613820) and short-rib thoracic dysplasia type 4 (OMIM 613819) (Davis et al. 2011). Slow IFT transport has been reported for *Ttc21b* mutant mice with concomitant dysregulation of the Sonic hedgehog (SHH) pathway (Tran et al. 2008), supporting a causal relationship of IFT slowing and the phenotype in the present patient.

The patient with *CLUAP1* mutations and Leber congenital amaurosis had severe visual impairment with vision limited to light perception by 6 wk of age without other systemic features (Soens et al. 2016), whereas the patient presented here has a pleiotropic developmental disorder with features including polydactyly, cutaneous syndactyly, oculomotor apraxia, respiratory dysfunction, obesity, and an abnormal cerebellar vermis. When last seen at 9 yr of age, he did not have retinal degeneration. It is likely that the effects on protein function of the missense mutations identified in these two individuals are distinct, leading to the different phenotypes. The p.(Leu273Phe) variant in the patient with Leber congenital amaurosis was in the coiled-coil domain; amino acids 187–308 of CLUAP1 are known to be important in homopolymer formation. The p.(Met113Arg) mutation reported here is located within the calponin homology domain of CLUAP1, which is known to be important for interaction with another IFT protein, IFT80 (Taschner et al. 2016), also implicated in ciliopathy disorders. Alternatively, the variable phenotypes seen in these two patients could be related to the effects of other unknown modifying genes.

We conclude that the *CLUAP1* variants identified here are pathogenic and cause a phenotype within the Joubert syndrome spectrum. This is based on the finding of the molar tooth sign, considered pathognomonic for Joubert syndrome, the previously demonstrated role of *CLUAP1* variants in Leber congenital amaurosis (a known ciliopathy), biallelic variants that are rare but are predicted to be pathogenic, and functional analysis showing decreased IFT in a validated *Xenopus* model system*.* Mutation scanning for patients with Joubert syndrome and related phenotypes should include *CLUAP1* as a candidate gene. The identification of additional affected patients is essential to identify the mutational and phenotypic heterogeneity of this disorder.

## METHODS

### Exome Sequencing

DNA was isolated from the proband and his parents’ whole blood using the salting-out method (QIAGEN), followed by phenol–chloroform extraction. Solution hybridization exome capture was performed using the Illumina TruSeq system (Illumina). Flow cell preparation and paired-end read sequencing were performed using the HiSeq 2000 sequencer (Vilboux et al. 2016) (Illumina). Image analyses and base-calling were performed as described (Vilboux et al. 2016). Reads were aligned to hg19, NCBI 37, using NovoAlign (Novocraft Technologies). Samples were sequenced to sufficient coverage such that at least 85% of the targeted exome was called with high-quality variant detection (reported as genotype at every callable position). Genotypes were called using only those sequence bases with Phred base qualities of at least Q20 using Most Probable Genotype (Teer et al. 2010) (MPG) and an MPG score of ≥10. Filters were applied using the VarSifter Next-Gen variant analysis software (Teer et al. 2012). Variants were filtered for nonsynonymous, splice-site, frameshift, and nonsense alterations. To filter for rare variants, the ClinSeq cohort (1001 control individuals) was used with an initial minor allele frequency (MAF) filter of <0.005. Results were analyzed using both recessive and dominant de novo models. Sanger sequencing was performed for confirmation of variants detected by exome sequencing. Primer sequences included CLUAP1_1F, ATC CCA GTT GTC AGC AGA GG; CLUAP1_1R, CCA AAT GCT GAT GAG CAC AA; CLUAP1_2F, TGC TTG GGG AAG TGT CTA TTT; CLUAP1_2R, TTT CAG ACA TGA GCC ACC AC. Identified variants were assessed in the ExAC data set of 65,000 individuals to assess their population frequency (Lek et al. 2016). Mutation nomenclature is according to Human Genome Variation Society (HGVS) standards using reference sequence NM_015041.2.

### Plasmids and Microinjections to *Xenopus* Embryos

The wild-type *CLUAP1* open reading frame was cloned from human cDNA and inserted into pCS10R-GFP vector. To generate GFP-tagged mutant *CLUAP1* constructs, mutagenesis was performed with a QuikChange II site-Direct Mutagenesis Kit (Agilent Technologies). Capped and polyadenylated mRNAs were synthesized via an mMESSAGE mMACHINE SP6 Transcription Kit (Life Technologies). Of note, 100 or 150 pg of GFP-CLUAP1 mRNA, 50 pg of cent4-RFP mRNA, and 50 pg mem-RFP mRNA were injected into two ventral blastomeres of four-cell stage embryos.

### Live Imaging and Analysis

Embryos at stage 25–28 were anesthetized with 0.005% benzocaine. Images were captured with a LSM 700 confocal microscope with an 63×/1.4 oil immersion objective (Carl Zeiss). High-speed in vivo images were acquired on a Nikon Eclipse Ti confocal microscope with a 63×/1.4 oil immersion objective. Time-lapse series were captured 266.5 msec per frame. Images were analyzed by ImageJ.

### Western Blotting

Embryos were lysed in M-PERTM Mammalian Protein Extraction Reagent (ThermoFisher Scientific) containing protease inhibitor cocktail (Sigma-Aldrich) and phenylmethylsulfonyl fluoride. Embryo extracts were applied to sodium dodecyl sulfate (SDS)–polyacrylamide gel electrophoresis (PAGE) gels and transferred onto nitrocellulose membrane. The GFP (1:2000 dilution, sc-9996, Santa Cruz) and actin (1:2000 dilution, ab1801, Abcam) antibodies were used as primary antibodies. The GFP or actin proteins were detected with horseradish peroxidase (HRP)-anti mouse or rabbit IgG antibody (1:10,000, Pierce or Jackson ImmunoResearch Laboratories, respectively) and visualized with the SuperSignal West Dura Extended Duration Substrate (Pierce).

## ADDITIONAL INFORMATION

### Data Deposition and Access

The sequencing data is available in the National Center for Biotechnology Information (NCBI) database of Genotypes and Phenotypes (dbGaP; https://www.ncbi.nlm.nih.gov/gap) under study number phs001348.v1.p1. Variants have been submitted to ClinVar (https://www.ncbi.nlm.nih.gov/clinvar/) under accession numbers SCV000299272 and SCV000299273.

### Ethics Statement

This study was performed under National Human Genome Research Institute (NHGRI) institutional review board (IRB)-approved protocols 10-HG-0065 and 94-HG-01903 that include approval for publication of results. Written informed consent was obtained according to those approved protocols.

### Acknowledgments

The authors gratefully acknowledge the family for their contributions to this work.

### Author Contributions

L.G.B., J.B.W., and J.M.G. designed and interpreted the studies. J.J.J. and I.M.W. performed and interpreted the human genomic analyses; C.L. performed and interpreted the animal model experiments. M.A.P., M.M.C., and J.C.S. performed the clinical analyses. J.J.J. wrote the initial draft of the manuscript, and all coauthors edited it for important scientific content.

### Funding

This work was supported by funding from the Intramural Research Program of the National Human Genome Research Institute (1 ZIA HG200328 11 and 1 ZIA HG200388 03 to L.G.B., J.J.J., M.M.C., J.C.S., and I.M.W.). C.L. and J.B.W. were supported by funding from the NHLBI (HL117164).

### Competing Interest Statement

L.G.B. is an uncompensated advisor to the Illumina Corp. and receives honoraria from Wiley-Blackwell. The other authors declare no competing interests.

### Referees

Peter N. Robinson

Anonymous
